# Mares Prefer the Voices of Highly Fertile Stallions

**DOI:** 10.1371/journal.pone.0118468

**Published:** 2015-02-25

**Authors:** Alban Lemasson, Kévin Remeuf, Marie Trabalon, Frédérique Cuir, Martine Hausberger

**Affiliations:** 1 Université de Rennes 1, Laboratoire d’éthologie animale et humaine, UMR 6552- C.N.R.S., Paimpont, France; 2 Institut Universitaire de France, Paris, France; 3 Haras du Pin, Le Pin-au-Haras, France; 4 C.N.R.S., Laboratoire d’éthologie animale et humaine, UMR 6552- Université de Rennes 1, Rennes, France; Utrecht University, NETHERLANDS

## Abstract

We investigated the possibility that stallion whinnies, known to encode caller size, also encoded information about caller arousal and fertility, and the reactions of mares in relation to type of voice. Voice acoustic features are correlated with arousal and reproduction success, the lower-pitched the stallion’s voice, the slower his heart beat and the higher his fertility. Females from three study groups preferred playbacks of low-pitched voices. Hence, females are attracted by frequencies encoding for large male size, calmness and high fertility. More work is needed to explore the relative importance of morpho-physiological features. Assortative mating may be involved as large females preferred voices of larger stallions. Our study contributes to basic and applied ongoing research on mammal reproduction, and questions the mechanisms used by females to detect males’ fertility.

## Introduction

Vocal communication is primordially important in the daily social life of a large number of mammal species. Just by hearing a given voice, some mammals, including humans, are able to assess their degree of familiarity with the caller (e.g. Campbell’s monkeys [1], Asian small-clawed otters [2]), its size (e.g. red deer [3], koalas [4], giant pandas [5]), and its identity (e.g. olive baboons [6], Atlantic walrus [7]). Auditory decoding capacities concerning internal characteristics of callers have also been demonstrated. For example, baboons [8] and spotted hyenas [9] encode acoustically the dominance rank of the caller. Humans can reliably estimate the age of a speaker just by his/her voice characteristics [10].

One of the main functions of vocal signals is to coordinate interactions between conspecifics. Among these interactions, exchange of vocal signals between conspecifics of opposite sexes is frequent and serves to attract new mates (e.g. mouse lemurs [11]), to strengthen male-female bonding (e.g. siamangs [12]), and to coordinate copulation or to inform about copulation success (e.g. Barbary macaques [13]). The application of the source—filter theory has enabled researchers to decompose the acoustic structure of vocal signals according to their mode of production and to predict the acoustic variation that is caused by anatomical or physiological attributes of the caller [14]. Hence, acoustic encoding of physiological characteristics of a potential sexual partner plays a role in the outcome of a given interaction. However, studies addressing this question remain limited, notably concerning mammals compared to birds and insects. For example, males discriminate the different stages of a female’s oestrous cycle by her voice (Barbary macaques [15], giant pandas [16], humans [17]). Hormonal, notably testosterone, levels, are correlated with frequency parameters (e.g. fundamental frequency, dominant frequency, frequency modulation rate) of male voices (e.g. humans [10, 18]), a trait that can impact females’ preference (e.g. giant pandas [19]). Frequency parameters predict reproduction success of the few species investigated (humans [20, 21], red deer [22], fallow deer [23]).

Horses are interesting models in this perspective for several reasons. First, male-female interactions play a key role in their social system. Horse populations are structured by long-lasting social and breeding relationships in polygynous groups [24]. The basic social unit of free-ranging horses is the harem composed mostly of two to six individuals, including adults (1–2 males, 2–3 females) who have established stable bonds, and juveniles (under 3 years old) [25, 26]. Physiologically, sexual maturity is reached at around 18 and 20 months old respectively in males and females [25, 26]. Juveniles of both sexes socially disperse, and adult males stay all year long with the females. Females express mate choice at two levels. Females emigrate from their natal group when they reach sexual maturity, dispersal peaking during the sexual receptivity season [27]. Females can create their own harem with a bachelor male or immigrate into an already formed harem [27]. Late female dispersal occurs sometimes when the stallion of a harem dies or when he is displaced [24]. Alternatively, females can accompany the migrating male. During their lifetime, females are not all equally receptive, older and more experienced mares are more receptive [28]. Authors have hypothesized that females’ libido and reproductive efficiency are related to the time spent in contact with a male [29]. Przewalski stallions can sometimes be rejected by mares that are dominant over them and hence fail to mate [30]. In the wild thus, females do express clear choices by first approaching a preferred stallion and then integrating his family group: visual attention, approach and proximity seeking are therefore key elements in the process of mate choice. In captivity, when presented a choice of males, females prefer some males to others [31, 32], but the question of how the choice is made remains open. Second, horses’ vocal repertoire includes a loud and long-distance call, named whinny, used for distant communication (up to 1 km) when visual and olfactory signals are not reliable. Whinnies are notably exchanged between or within sexes when spatially or visually separated [25, 36]. Whinnies encode caller’s sex, size and potentially dominance status [33]. Playback experiments showed that horses use whinnies to decode their degree of familiarity with the caller [33], as well as its identity [34]. Third, these large mammals can be handled easily by human experimenters enabling the collection of a variety of physiological parameters. Fourth, understanding the mechanisms facilitating the control of the reproduction of this domestic species is of primary importance for stud farms and breeders [35].

This study asked whether stallion voices encode their physiological characteristics, and whether mares display preferences for certain types of voice. We investigated relationships between acoustic characteristics, notably frequency-related parameters that have been showed to be crucial [36], and physiological features (i.e. heart beat, hormonal profile, sperm quality and reproductive success) known to be involved in arousal (i.e. motivational and emotional states) and fertility (Study 1). We hypothesized that if, as predicted, pitch is of primal importance, females’ auditory preferences can be evaluated by playback experiments (Study 2—Experiment 1). As pitch is already known to encode stallion size [33], we evaluated the influence of females’ size on the strength of the above preference (Study 2—Experiment 2). This relationship is predicted by the hypothesis of phenotypic assortative mating [37], a nonrandom mating pattern in which individuals with similar phenotypes mate with one another more frequently. Assortative mating has been proposed as an adaptive process to favour local populations with local adaptations to environmental conditions. Wild populations of horses do show local adaptations in terms of morphology and size therefore this possibility seemed worth testing. Moreover, experience may play a role and domestic mares are generally mated with a male of assorted breed hence size. Here, we predicted that taller mares should be more attracted by tall stallions than smaller mares.

## Study 1: Acoustic Encoding of Physiological Features by Stallions

### Materials and methods

#### Ethics statements

Studies 1 and 2 were performed in accordance with the routine procedures in the facilities where stallions are handled daily and regularly submitted to blood sampling. All procedures other than recordings and observations were performed by farm staff under the supervision of the local veterinarian. Moreover, experiments (in studies 1 and 2) complied with the current French (Centre National de la Recherche Scientifique) regulations governing the care and use of research animals and have been approved by the French National Institute for Horse and Horse riding (Institut français du cheval et de l’équitation). Experiments were performed in accordance to the 2010/63/EU European Communities Council Directive. The stud farm staff was responsible for all animal husbandry and care. As our experiments were based on non-invasive observations no ethical approval was required.

#### Subjects

In February 2012, we recorded the calls and heart beats of 15 breeding stallions (S1–S15) of various breeds and ages, housed on three different French stud farms (Table 1). These stallions were housed individually in 3m x 3m stalls, with a natural photoperiod, and were moved to paddocks for one or two hours each day. Horses were fed hay and pellets twice a day. Water was provided *ad libitum*. These stallions were handled every year for sperm collection.

**Table 1:** Stallion’s characteristics.

Stallion #	Age (y)	Breed	Stud farm
S1	15	Holsteiner	Saint-Lô
S2	17	Thoroughbred	Le Pin
S3	27	Dutch warmblood	Lamballe
S4	17	French trotter	Le Pin
S5	23	Arab	Lamballe
S6	13	French saddlebred	Saint-Lô
S7	13	French saddlebred	Le Pin
S8	12	French trotter	Le Pin
S9	10	French trotter	Le Pin
S10	10	French saddlebred	Saint-Lô
S11	10	French trotter	Lamballe
S12	9	French saddlebred	Saint-Lô
S13	9	Arab	Le Pin
S14	14	Saddle horse of foreign origin	Le Pin
S15	4	Connemara	Saint-Lô

#### Acoustic recordings and measurements

Stallions’ whinnies were recorded with a directional microphone (K6/ME66 Sennheiser) connected to a digital portable recorder (PMD661 Marantz, sampling rate 44100Hz, resolution 16 bits). Each stallion was led outdoors twice by one caretaker for 10 min recordings, on different days, in a random order. During a recording session, the male stood 50m from his stall and another caretaker led a familiar mare (the same for all males on a stud farm) keeping 10–20m away. The experimenter (K.R.) stood 5m in front of the male with the recording apparatus. All males were recorded in the same context (excitement-frustration) to avoid situation-specific acoustic variations. A total of 178 whinnies (12 ± 6 per subject, range: 10–18) were recorded and used for the subsequent analyses.

**Fig 1:**
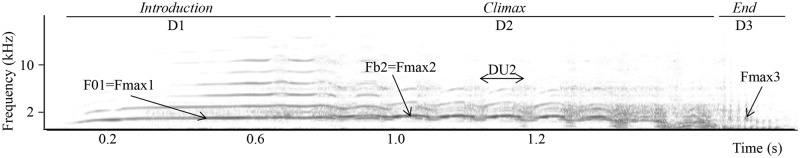
A spectrogram of a whinny showing acoustic measures taken.

Spectrograms were extracted using ANA software [38] with Linux (FFT size 256) for subsequent acoustic measurements. A typical whinny is composed of three parts [33]: a first loud, relatively flat and tonal part (named here Part 1 or ‘Introduction’), a second relatively atonal part, more or less rhythmical, including long modulated units (Part 2, ‘Climax’) and a third very soft and rhythmical part with very short units (Part 3, ‘End’). For comparative reasons, we measured the same 12 acoustic parameters as Lemasson et al. [33] and averaged the data per stallion (Fig. 1): F01 (fundamental frequency of the Introduction), Fmax1, 2, 3, T (dominant frequency, i.e. frequency value with the maximal intensity, of Parts 1, 2, 3 and entire call, measured on the intensity spectrum), Fb2 (basic frequency of the Climax, i.e. first frequency reinforced, because the fundamental frequency is not always identifiable), D1, 2, 3, T, U2 (durations of Parts 1, 2, 3, entire call and Part 2 units), NbU2 (number of units in Part 2).

#### Heart beat and hormonal measuring

Before being led outdoors for acoustic recording, subjects were equipped with a heart rate monitor for horses (Polar Horse Trainer S810i). With this equipment, the experimenter could record heart beat within a 5 second window during which a whinny was emitted (HB5s) and during the 10 min of the recording session (HB10m). HB5s and HB10m were averaged per male for subsequent comparisons.

The same week, a caretaker (the same for all stallions at each location) familiar to the horses, collected two blood samples per handled subject, on two consecutive days, using 4ml blood collection tubes BD Vacutainer Lithium Heparin 68. Samples were collected from the jugular vein between 9 and 10 am, within 1–2 minutes, when stallions were calm and still in stalls. The blood sampling site was first swabbed with alcohol. Only one 4ml blood tube was collected per horse and per day to sample the minimum necessary for analyses. Plasma was immediately separated by centrifugation at 4°C (15 min at 3000 rpm) and the aliquot extracts were stored at-36°C until assayed (cortisol and testosterone, as we were interested in respectively emotion- and reproduction-related hormones). Hormone concentrations were measured without an extraction procedure, using a commercially available EIA kit and performed according to the manufacturer’s guidelines (Assay Designs Inc., USA). The concentrations of hormones in plasma samples were calculated from a standard curve and expressed in pg/ml. The two concentrations for each hormone were averaged per subject. The intra- and inter-assay coefficients of variation were below 5.4% and 10.9% respectively for cortisol, 7.8% and 12.6% respectively for testosterone.

#### Reproductive success and semen features

Data on previous breeding records for the different stallions were made available by the National studs. Hence, we could calculate two reproductive success scores for all stallions [35]: Insemination Success (IS = number of embryos alive after 16 days / number of artificial inseminations) and Survival Success (SS = number of pregnant mares at the end of the gestation / number of embryos alive after 16 days). This was based on 294 ± 176 artificial inseminations per stallion performed between 2007 and 2012.

Spermograms by stud farms between 2006 and 2012 for all stallions (except horse # S1 for which spectrograms were not available) enabled the scoring of sperm mobility at 0h (M0), 24h (M24) and 48h (M48) after ejaculation, as well as the percentage of spermatozoa presenting physical abnormalities in the mid-piece in particular (SpM), or in all parts (SpO). Spermograms were done using Palmer and Fauquenot’s protocol [39].

#### Statistical analyses

Given our small sample sizes (N = 14 for sperm-related variables and N = 15 for all other cases), we used nonparametric Spearman tests to assess correlations between physiological and acoustical parameters. Statistics were run on Statistica 8.0 (Statsoft) with a significance threshold of 0.05.

### Results

Physiological and reproductive profiles differed among stallions (Table 2). However, reproductive success was consistent between insemination (IS) and survival success (SS) data (Table 3). Similarly, different measures of sperm mobility (M0, M24, M48 altogether), of spermatozoon abnormality (SpM with SpO) and of heart rates (HB5s with HB10m) were positively correlated. Heart beat rates were negatively correlated with one measure of fertility (HB10m/SS). Sperm mobility and spermatozoon abnormalities (M0/SpM, M0/SpO, M48/SpM, M48/SpO) were negatively correlated. However, hormonal levels (testosterone and cortisol) were not correlated with any other parameter.

**Table 2:** Acoustic (a) and physiological (b) traits of stallions (mean ± SE) (N = 15 stallions).

(a)	Mean	Standard error	(b)	Mean	Standard error
DT (ms)	1898	69	HB10m (/min)	93.93	3.78
FmaxT (Hz)	1229	102	HB5s (/min)	96.04	4.2
D1 (ms)	524	41	Testosterone (pg/mL)	1.82	0.27
F01 (Hz)	700	54	Cortisol (pg/mL)	44.91	3.54
Fmax1 (ms)	1334	73	IS	0.52	0.01
D2 (ms)	1065	53	SS	0.79	0.01
Fmax2 (Hz)	1197	91	M0 (%)	69.7	3.48
Fb2 (Hz)	625	45	M24 (%)	55.02	4.48
NbU2	7.44	1.04	M48 (%)	37.64	5.54
DU2 (ms)	327	96	SpM (%)	17.79	2.4
D3 (ms)	311	22	SpO (%)	30.14	2.97
Fmax3 (Hz)	912	78			

See text for signification of abbreviations.

**Table 3:** Relationships among physiological features.

	HB5s	IS	SS	Testosterone	Cortisol	M0	M24	M48	SpM	SpO
**HB10m**	**15**	15	**15**	15	15	14	14	14	14	14
	**0.55**	-0.148	**-0.534**	0.261	-0.116	-0.162	0.279	0.057	0.158	0.166
	**0.033**	0.598	**0.04**	0.348	0.68	0.581	0.335	0.846	0.589	0.571
**HB5s**		15	15	15	15	14	14	14	14	14
		-0.057	-0.368	0.404	0.211	0.358	-0.482	0.389	-0.16	-0.312
		0.84	0.177	0.138	0.451	0.209	0.081	0.169	0.584	0.278
**IS**			**15**	15	15	14	14	14	14	14
			**0.568**	-0.325	-0.175	-0.128	0.187	0.114	-0.029	0.128
			**0.027**	0.237	0.533	0.662	0.522	0.697	0.314	0.662
**SS**				15	15	14	14	14	14	14
				-0.429	-0.047	-0.06	-0.154	0.099	0.147	0.303
				0.111	0.869	0.84	0.599	0.736	0.615	0.293
**Testosterone**					15	14	14	14	14	14
					0.293	0.172	0.339	-0.011	-0.13	-0.17
					0.289	0.556	0.236	0.97	0.659	0.561
**Cortisol**						14	14	14	14	14
						0.064	0.02	-0.132	0.376	0.133
						0.828	0.946	0.653	0.185	0.651
**M0**							**14**	**14**	**14**	**14**
							**0.606**	**0.758**	**-0.685**	**-0.812**
							**0.022**	**0.002**	**0.007**	**0**
**M24**								**14**	14	14
								**0.59**	-0.365	-0.416
								**0.026**	0.199	0.139
**M48**									**14**	**14**
									**-0.73**	**-0.704**
									**0.003**	**0.005**
**SpM**										**14**
										**0.919**
										**0**

Results of Spearman tests are given in each cell: upper value = N, intermediary value = rs, lower value = p (bold type: significant correlations).

**Fig 2:**
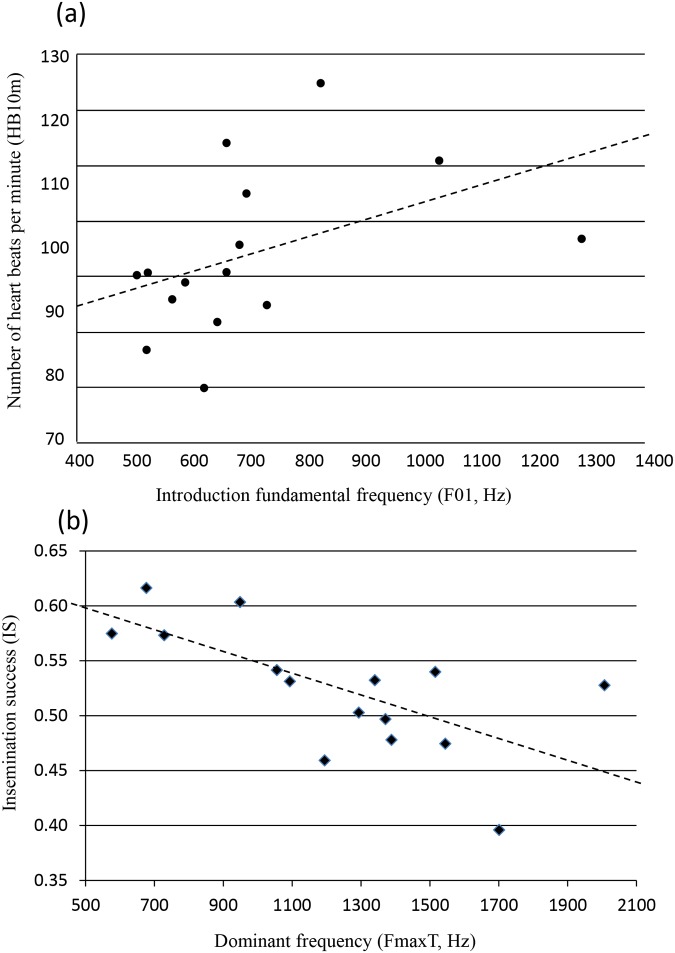
Correlation between voice frequencies in relation to (a) heart beat rates (P = 0.03) and (b) fertility scores (P = 0.002).

**Fig 3:**
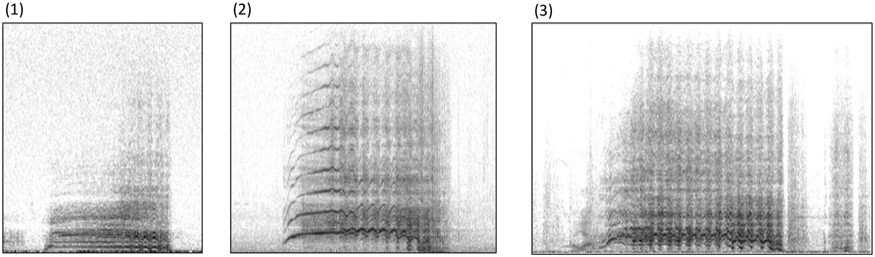
Heart beat rates in relation to number of repeated units in the climax of the whinny. Example of three spectrograms (X axis = duration, Y axis = frequency): (1) NBU2 = 4—HB5s = 174 beats per minute (bpm), (2) NBU2 = 8—HB5s = 112 bpm, (3) NBU2 = 15—HB5s = 73 bpm.

Acoustic parameters were correlated with heart beat rates and fertility, but with no other physiological parameter (Table 4). The most important parameter appeared to be the pitch of the stallion’s voice. The lower-pitched the stallion’s voice, the slower its heart beat (positive correlations: FmaxT/HB5s, Fmax2/HB5s, F01/HB10m, Fb2/HB10m; Fig. 2a), and the higher its fertility (negative correlations: FmaxT/IS and SS, F01/IS, Fmax1/IS and SS, Fmax2/IS and SS; Fig. 2b). One temporal acoustic parameter was also correlated with physiology: the higher the number of repeated units in the climax (Fig. 3), the slower the heart beat (NBU2/HB5s).

**Table 4:** Relationships between acoustic and physiological features.

	Heart beat		Reproductive success		Sperm mobility			Spermatozoa abnormality		Testosterone	Cortisol
	HB10m	HB5s	IS	SS	M0	M24	M48	SpM	SpO		
**DT**	15	15	15	15	14	14	14	14	14	15	15
	-0.291	-0.011	-0.314	0.064	0.06	-0.257	0.073	-0.103	-0.176	0.029	0.025
	0.292	0.97	0.254	0.82	0.84	0.374	0.805	0.725	0.551	0.92	0.93
**FmaxT**	15	**15**	**15**	**15**	14	14	14	14	14	15	15
	0.449	**0.514**	**-0.739**	**-0.693**	-0.024	-0.068	-0.196	0.156	-0.011	0.254	0.107
	0.093	**0.05**	**0.002**	**0.004**	0.934	0.817	0.502	0.594	0.97	0.362	0.704
**D1**	15	15	15	15	14	14	14	14	14	15	15
	-0.084	0.271	-0.15	-0.246	0.197	-0.042	0.112	-0.073	-0.276	-0.036	0.071
	0.766	0.328	0.594	0.376	0.5	0.888	0.703	0.805	0.339	0.899	0.8
**F01**	**15**	15	**15**	15	14	14	14	14	14	15	15
	**0.573**	0.322	**-0.515**	-0.498	0.149	0.246	0.04	-0.002	-0.119	0.27	-0.148
	**0.03**	0.242	**0.05**	0.058	0.61	0.397	0.893	0.994	0.684	0.331	0.598
**Fmax1**	15	15	**15**	**15**	14	14	14	14	14	15	15
	-0.261	-0.379	**-0.636**	**-0.604**	-0.046	-0.143	-0.378	0.253	-0.075	0.154	0.107
	0.346	0.164	**0.011**	**0.018**	0.875	0.626	0.182	0.383	0.798	0.585	0.704
**D2**	15	15	15	15	14	14	14	14	14	15	15
	-0.261	-0.361	-0.186	0.221	-0.165	-0.224	-0.145	-0.02	0.004	-0.289	-0.204
	0.346	0.187	0.508	0.428	0.597	0.441	0.62	0.946	0.988	0.296	0.467
**Fmax2**	15	**15**	**15**	**15**	14	14	14	14	14	15	15
	0.504	**0.546**	**-0.657**	**-0.618**	-0.051	-0.07	-0.233	0.24	0.08	0.214	0.136
	0.554	**0.035**	**0.008**	**0.014**	0.863	0.811	0.422	0.409	0.787	0.443	0.63
**Fb2**	**15**	15	15	15	14	14	14	14	14	15	15
	**0.577**	0.413	-0.492	-0.4	0.375	-0.184	0.446	-0.339	-0.368	0.311	-0.095
	**0.024**	0.126	0.063	0.139	0.186	0.529	0.11	0.236	0.204	0.259	0.737
**NbU2**	15	**15**	15	15	14	14	14	14	14	15	15
	-0.335	**-0.543**	-0.214	0.213	-0.076	-0.249	-0.021	0.141	0.11	-0.354	0.141
	0.223	**0.036**	0.443	0.447	0.795	0.391	0.943	0.631	0.709	0.225	0.616
**DU2**	15	15	15	15	14	14	14	14	14	15	15
	-0.093	-0.032	0.304	-0.079	-0.183	-0.11	-0.431	0.16	0.071	0.229	0.021
	0.742	0.909	0.271	0.781	0.53	0.708	0.124	0.584	0.81	0.413	0.94
**D3**	15	15	15	15	14	14	14	14	14	15	15
	-0.257	0.182	-0.05	-0.004	-0.36	-0.264	-0.207	-0.081	0.009	0.257	0.193
	0.354	0.516	0.86	0.99	0.206	0.362	0.478	0.782	0.976	0.355	0.491
**Fmax3**	15	15	15	15	14	14	14	14	14	15	15
	0.266	0.179	-0.404	-0.336	0.049	0.4	-0.057	0.182	-0.176	0.364	0.179
	0.337	0.524	0.136	0.221	869	0.156	0.846	0.533	0.563	0.182	0.524

Results of Spearman tests are given in each cell: upper value = N, intermediary value = rs, lower value = p (bold type: significant correlations, i.e. ≤ 0.05).

## Study 2: Auditory Preference of Mares

### Materials and methods

#### Subjects

Playback subjects were 40 adult (7 to 27 years old) mares. Group A (N = 14, 16.5 +/- 4.8 years old) and B (N = 15, 16 +/- 5.6 year old) females of similar height (165 +/- 5 cm), were subjects of Experiment 1 and were housed at ‘Haras National du Pin’ (National stud, France). Sizes of group C (N = 11, Age 11.5 +/- 1.9) females were very heterogeneous (155 +/- 11 cm tall). They were the Experiment 2 subjects and were housed at Ploermel riding school (France) (Table 5). The group A and B mares had known one another for more than 3 years and were housed outdoors in their respective enclosures (300m^2^), with an *ad libitum* access to shelters and water. The group C mares were housed individually in their own 12m^2^ stalls of the riding school. All mares were fed hay and pellets once a day. Water was available *ad libitum*. All females were unfamiliar to the stallions (housed in a different stud) used in these experiments. All group A and B females have been inseminated at least once. Fourteen of them had experienced giving birth. We have no information concerning the experience of females from the three groups of natural covering by stallions.

**Table 5:** Mares’ characteristics.

Mare #	Age(yo)	Size(cm)	Breed	Group #
M1	13	172	SF	A
M2*	13	162	SF	A
M3°	27	157	TF	A
M4	16	160	SF	A
M5	11	157	AA	A
M6°	22	159	AA	A
M7*	15	167	SF	A
M8*	12	160	AA	A
M9°	22	157	SF	A
M10*	20	165	SF	A
M11	12	170	AA	A
M12*	19	168	SF	A
M13*	15	172	SF	A
M14	14	167	SF	A
M15°	21	158	SF	B
M16	18	172	SF	B
M17°	17	163	SF	B
M18°	14	166	TF	B
M19*	20	167	SF	B
M20*	7	162	TF	B
M21	11	165	AA	B
M22	20	166	SF	B
M23*	14	161	TF	B
M24	18	165	TF	B
M25	14	173	SF	B
M26*	19	170	SF	B
M27	18	162	SF	B
M28*	12	Not available	SF	B
M29	17	166	SF	B
M30	7	139	KWPN	C
M31	22	155	SF	C
M32	17	160	SF	C
M33	13	138	COxTR	C
M34	13	158	SF	C
M35	13	170	SF	C
M36	12	164	SF	C
M37	8	167	SF	C
M38	7	154	SF	C
M39	6	144	PFS	C
M40	8	151	PS	C

The size corresponds to the horse withers height. Estrus and anestrus females are respectively marked with * and °.

Size: withers height. Females in estrus are marked with an asterisk.

Breeds: SF = French saddlebred; TR = French trotter; AA = Anglo-Arabian; KWPN = Dutch warmblood; CO = Connemara; PFS = French riding pony; PS = Thoroughbred.

#### Experiment 1

Prior to playback experiments, group A and B mares had been hormonally treated with Regumate (altrenogest) to try to synchronize their cycles and to increase chances to be in estrus at the time of experiments. The day before and the day after Experiment 1, each female was presented to a stallion, separated by a wooden board, to assess their sexual receptivity following the Asa’s protocol [40]. Females that were receptive before and after playbacks were considered (in this study) to be in oestrus state (N = 11), whereas females that were not receptive before and after playbacks were considered in anoestrus state (N = 6). All other females presented an unstable status (receptive either before or after the experiment) and were thus not included in this comparison.

Playback experiments aimed at evaluating mares’ preferred pitch of a male voice. Each study group heard the whinnies of one pair of stallions (Group A—stallions S10 and S5, Group B—stallions S3 and S6). Stallions S10 and S3 had low pitched voices (their mean FmaxT were respectively 676 and 948 Hz) and stallions S5 and S6 had high pitched voices (their mean FmaxT were respectively 2007 and 1388 Hz).

**Fig 4:**
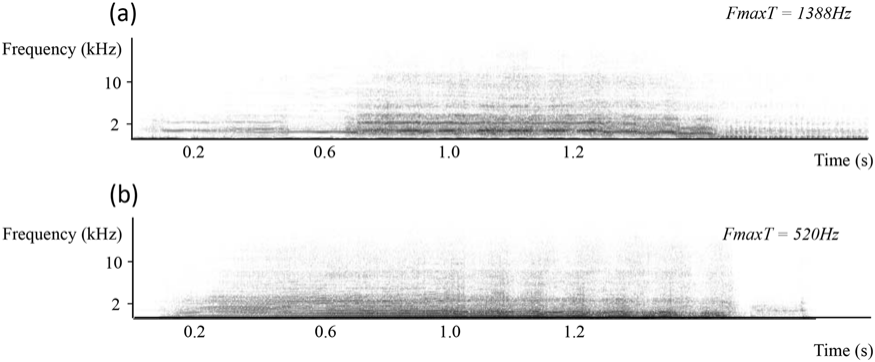
Acoustic stimuli spectrograms (a) low pitch and (b) high pitch.

Four whinny exemplars per stallion were used in the experiment. These exemplars were selected so that their FmaxT values were close to the corresponding stallion’s mean. During a given playback trial, a single exemplar of two whinnies (one Low pitched and one High pitched, see Fig. 4 and S1–S4 Sounds for examples) were broadcast simultaneously. Whinnies were paired so that their pitches differed but their durations matched (1948 +/- 109 ms). All whinnies were homogenized in amplitude so that they reached 70db in the centre of the experimental setup, a sound level matching natural conditions [33].

**Fig 5:**
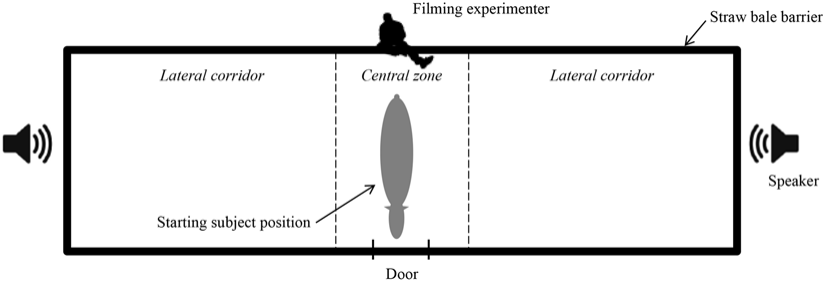
Playback experiment set up.

The experimental setup was a visually enclosed rectangle (3m x 10m) with a border of piled straw bales (3m high) (Fig. 5). The rectangle was divided virtually into three parts: a 2m-long central zone where females were placed, and two 4m-long lateral corridors. We placed a loudspeaker (JLH-2002 Sanha) at the end of each corridor, one for the low pitched stimulus and the other one for the high pitched stimulus. Loudspeakers were monitored wireless with a M51V Asus computer. The experimenter (K.R.) stayed still in the central zone filming the subject with a HDR-XRI55E Sony camera.

Each subject was familiarized with the setup the week preceding the experiment (i.e. females were walked individually all along the setup and then were freed for exploration twice for five minutes each time). The experiment lasted 4 consecutive days for each group (A: 16–19 April 2012, B: 23–26 April 2012), totalizing 8 trials per subject (one every morning between 8 and 11 am and one every afternoon between 2 and 5 pm). During a trial, a caretaker led a subject into the central zone, waited one or two minutes for the mare to calm down, adjusted her body position and freed the mare. The experimenter played the sounds when the mare’s body was strictly perpendicular to both loudspeakers with the mare facing the exit door and then filmed the female’s response for 5 minutes. Between two consecutive trials, a caretaker removed any dung and sprayed Desogerme (ethanol) to neutralize as much as possible the odours in the setup. A different pair of whinnies was played each day. The high pitched stimulus was broadcast from one side (randomly chosen) in the morning and from the other side in the afternoon so that each subject heard the same whinny from the left and the right on the same day.

The video recordings allowed quantification (by K. R.) of several behavioural items using the focal sampling method. We calculated the occurrence frequencies of ‘turn head towards High/Low pitched side’ (orientation of the head by more than 45° towards the loudspeaker) and of ‘turn body towards High/Low pitched side’. We recorded the total duration spent in each corridor of the setup as well as the first head orientation towards each loudspeaker. Data were coded blind as videos were scored in a random order with the sound off.

#### Experiment 2

The group C mares were not treated hormonally (and presented no clear sign of estrus) and each female could hear the same voices of the two stallions (S5 and S10) used in Experiment 1. These females were tested in a familiar 40m x 20m arena. Females were led to the arena and positioned in the centre following exactly the same procedure as in Experiment 1. Each mare was tested twice, at a 4-day interval. Tests were performed at 5–6 pm or 8–9 pm in a random order outside the riding centre’s activities. The same behaviours as in Experiment 1 were quantified for each 5-minute trial. Group C, including tall and short females, was suitable to test the assortative mating hypothesis. A standard criterion discriminating tall from small mares is withers height: above 151cm mares are called tall (French National Studbook).

#### Statistical analyses

Nonparametric Wilcoxon tests compared behavioural data of females from respectively groups A, B and C in relation to pitch of stimulus (Low *vs* High). Mann-Whitney tests compared total numbers of head and body orientations (regardless of side), as well as total durations in both corridors were between females oestrus and anoestrus states. We estimated the strength of preference for type of voice by calculating the following ratio: number of head orientations towards the Low-pitched voice / number of head orientations towards the high-pitched voice. We compared the ratio of mares in oestrus *vs* anoestrus states (Experiment 1) and tall *vs* short mares (Experiment 2) with Mann-Whitney tests. Statistics were run on Statistica 8.0 (Statsoft) with a significance threshold of 0.05.

### Results

#### Experiment 1

Data for both groups (A and B) of mares showed that they spent significantly more time in the corridor where the low-pitched voice was played back than in the corridor with the high-pitched voice (Table 6). Females displayed significantly more first head orientations, total head orientations and total body orientations towards the loudspeaker with the low-pitched voice. All the above-mentioned variables, except total body orientations, remained significant for group B mares. However, only the number of first head orientations remained significant for group A mares. See S1 and S2 Videos for examples of immediate responses.

**Table 6:** Mares’ responses to playbacks (Experiment 1).

	Group	Low-pitched side	High-pitched side	N	T	Z	P
Number of first head orientations	A	6.07 +/- 0.32	1.93 +/- 0.32	14	**2.500**	**3.139**	**0.002**
	B	6.13 +/- 0.29	1.86 +/- 0.29	15	**0.000**	**3.408**	**0.001**
	A+B	6.10 +/- 0.21	1.90 +/- 0.21	29	**5.500**	**4.584**	**0.000**
Total number of head orientations	A	59.79 +/- 3.47	51.29 +/- 3.49	14	23.000	1.852	0.064
	B	101.33 +/- 6.08	66.27 +/- 5.13	15	**0.000**	**3.408**	**0.001**
	A+B	81.28 +/- 5.26	59.03 +/- 3.40	29	**31.000**	**4.033**	**<0.001**
Total number of body orientations	A	22.86 +/- 2.55	19.93 +/- 2.06	14	31.500	1.318	0.187
	B	28.53 +/- 2.94	23.73 +/- 2.43	15	33.500	1.505	0.132
	A+B	25.79 +/- 1.99	21.90 +/- 1.61	29	**123.000**	**2.043**	**0.041**
Time spent in each corridor (s)	A	539.18 +/- 38.48	484.47 +/- 35.46	14	27.000	1.601	0.109
	B	543.03 +/- 51.24	426.89 +/- 34.05	15	**13.000**	**2.669**	**0.008**
	A+B	541.17 +/- 31.80	454.69 +/- 24.73	29	**69.000**	**3.211**	**0.001**

Low/High-pitched side columns: means +/- standard errors

T/Z/P: results of Wilcoxon matched-pairs signed-ranks tests (bold type: significant differences).

Whether mares were or not in estrus did not influence the strength of the preference for one type of voice (ratio of head orientations towards Low- / High-pitched voices, Mann-Whitney test, N_1_ = 11, N_2_ = 6, U = 32, P = 0.919). It also did not influence the number of head (U = 16, P = 0.088) and body (U = 23.5, P = 0.338) orientations. However, females in anoestrus state spent longer (1227 +/- 47 s) in the corridors broadcasting both low- and high-pitched voices than did estrus females (981 +/- 90 s) (U = 11, P = 0.027).

#### Experiment 2

Group C mares displayed significantly more head and body orientations towards, and spent significantly longer in the corridor with low-pitched voice than the other corridor (Table 7). However, numbers of first head orientations did not differ between the two types of stimuli.

**Table 7:** Mares’ responses to playbacks (Experiment 2).

Group C (N = 11)	Low-pitched side	High-pitched side	T	Z	P
Number of first head orientations	1.55 +/- 0.21	0.45 +/- 0.21	4.500	1.890	0.059
Total number of head orientations	20.91 +/- 2.03	11.09 +/- 1.28	**1.500**	**2.801**	**0.005**
Total number of body orientations	7.91 +/- 0.87	6.45 +/- 0.76	**6.500**	**2.141**	**0.032**
Time spent in each corridor (s)	187.30 +/- 33.07	105.73 +/- 24.14	**11.000**	**1.956**	**0.050**

Low/High-pitched side columns: means +/- standard errors.

T/Z/P: results of Wilcoxon tests (bold type: significant differences, i.e. ≤ 0.05).

**Fig 6:**
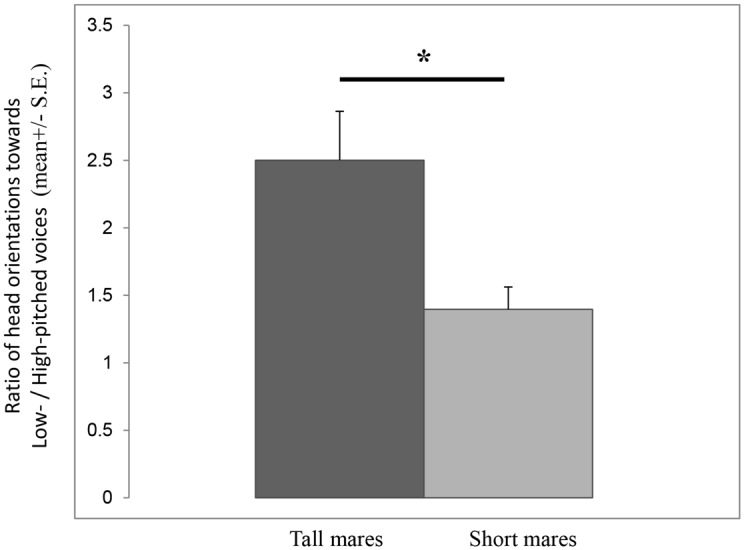
Group C mares’ preference for low-pitched voices in relation with their size: tall (N = 7), short (N = 4). *Result of Mann-Whitney test: U = 3, P = 0.038.

This group included seven ‘tall’ (“horse” size) and four ‘small’ (“pony” size) mares (see methods for definition). In line with the assortative matching prediction, and knowing that taller stallions possess lower pitched voices [33], the preference for low-pitched voices was significantly higher in tall than in small females (Fig. 6).

### Discussion

Our analyses of stallions’ voice acoustic features revealed that some parameters, essentially frequency-related measures, were correlated to fertility (survival success of embryos) as well as heart rate; the lower-pitched the stallion’s voice (fundamental and dominant frequency), the slower its heart beat and the higher its reproductive success (Study 1). No correlation was found between hormonal profile or sperm quality and any other physiological and acoustic parameters. Playback experiments revealed that females from our three studied groups are able to discriminate low- and high-pitched voices, and are more attracted by males displaying low-pitched voices (assessed by the time spent near the corresponding loudspeaker) (Study 2). Whether mares were or not in oestrus state did not influence the strength of their preference for one type of voice, suggesting that the above preference is not systematically related to mating or fertility (Experiment 1), as can be expected in social groups when males and females establish long-term stable bonds. Indeed, directing its attention and interest towards a particular male is the first indispensable step for an active choice of partner but not a direct proof of sexual motivation. Supporting the assortative mating prediction [37], the strength of the preference for low-pitched voices of tall females was higher than that of small females (Experiment 2).

Females’ preference for low pitched voices, as found here in mares, is a widespread characteristic. Attractiveness of human voices varies and listeners largely agree on which sounds are attractive (reviewed by McDermott [36]). Occidental woman, more so during the fertile phase [41], judge low-pitched male voices more attractive [42, 43, 44, 45]. Females of various mammal species are also more attracted by voices with lower pitched frequencies (red deer [3], koalas [4], giant pandas [5]). The attractiveness of males with low-pitched voices is also confirmed by studies showing related higher mating success (humans [46], bison [47]).

According to some authors, the main reason why low-pitched voices are found attractive is that frequencies are often negatively correlated with male size and/or weight and thus fighting/protecting abilities (red deer [22], koalas [4], giant pandas [48], otters [49], killer whales [50], bison [47], humans [10]). In line with this, our previous study showed that fundamental or dominant frequencies and stallion size were negatively correlated [33]. Women also relate low-pitched voices to masculinity, hard working ability and leadership [51, 52]. However, the relation between absolute frequency measure (e.g. fundamental or dominant frequency) and body size is not universal as sometimes spacing between formants is a better predictor of the emitter size in mammals (macaques [53]).

Besides protection abilities, female preference for low-pitched voices can be related to other male characteristics such as reproduction qualities. Our study showed that frequency-related voice features are reliable predictors of male reproduction success. Most reports show that fundamental and dominant frequencies are key acoustic features carrying sexual information. For example, the dominant frequency (e.g. Barbary macaques [15]) and the harshness (i.e. atonality) of frequency distribution (e.g. humans [17]) vary throughout the oestrous cycle in females. Female human voices during menstruation appear less attractive to men [17]. Several studies confirm the link between frequencies and fertility. Female high-pitched voices [20] and male low-pitched voices [21] predict higher infant survival rates respectively for indigenous Namibians and Tanzanian hunter-gatherers. The fundamental frequency of male red deer voices was positively correlated with embryo survival rates [22].

However, our study also questions the relevance of different physiological parameters for assessing male reproduction qualities. Here, male reproduction success (measured by artificial inseminations) was not related to sperm quality or hormonal profile. On one hand, this contradicts previous reports showing a correlation between reproduction success of male wild mammals and sperm quality (red deer [54], Mexican gray wolves [55]). On the other hand, the correlation, intensively studied, between semen quality, notably mobility and morphological abnormalities, and fertility of domestic mammals is highly controversial and subject to great inter-study variations [56, 57], notably for horses (reviewed by Graham [35]). Testosterone concentrations and fertility of red deer were not correlated [54]. Humans’ probability of conception increases with increased sperm concentration, but only up to a certain threshold. However semen quantity and quality were not good predictors of pregnancy for Danish couples [58]. As in our study, other authors found no correlations or only limited correlations between voice pitch and sperm quality or quantity (e.g. humans [44]) or testosterone concentrations [36]. Nevertheless, we must acknowledge that semen and acoustic collections were not temporally synchronized in our study which may have played a role in the absence of correlation.

Interestingly, our data revealed a correlation between reproduction success and male calmness. Based on this study (reproduction success, heart beat) and our previous report (size: [33]), we can say that mares prefer the voices of taller stallions with lower heart beat rates and higher fertility. Lower heart beat rates are found in calmer situations and in calmer individuals. Previous studies confirmed that stressful events trigger increase in heart rates (dog [59], horses [60, 61]). The relative influence of these different male characteristics (fertility, size, arousal) on mares’ preferences remains open to debate. However, experiment 2 data indicate that at least stallion size matters, as the preference for low-pitched voices was stronger in taller than in smaller mares. This is an intriguing but interesting finding that deserves further investigation with a larger number of males with different sizes. One possible interpretation suggests that females make choices following an assortative mating / pairing pattern, as do humpback whales [62] and humans [42] for size and fallow deer for age [63]). Another possibility is that previous experience with same breed stallions may have created a memory in these mares, although at least some of them were never mated. Unfortunately, the life history was not known for all these adult females. Although we tested four males and four calls per male, we cannot totally exclude any influence of pseudoreplication. Replicating this playback experiment with larger numbers of males and calls per male would be necessary to control it.

### Conclusions

Our study highlights the importance of vocal communication in a species better known as a visual communicant [25]. Stallion acoustic signals may play a role in female attraction at the time of group formation, as well as a role in sexual stimulation or libido maintenance when a group is formed. This supports preliminary findings showing that (1) females base their approach preference towards certain unfamiliar males on the amount of vocal signals uttered during their first encounter [31]; and (2) some male vocal signals trigger mare behavioural preparation for mating (e.g. tail lifting, leg parting) [64, 65]. Our data also confirm that not all male voices are equally attractive [66], opening new lines of bioacoustic research on mammal reproduction.

## Supporting Information

S1 Sound Example of high-pitched acoustic stimulus. (WAV)

S2 Sound Example of high-pitched acoustic stimulus. (WAV)

S3 Sound Example of low-pitched acoustic stimulus. (WAV)

S4 Sound Example of low-pitched acoustic stimulus. (WAV)

S1 Video Immediate response in trials with a low-pitched sound on the right. (WMV)

S2 Video Immediate response in trials with a low-pitched sound on the left. (WMV)
